# Computational and experimental studies on the micellar morphology and emission mechanisms of AIE and H-bonding fluorescent composites†

**DOI:** 10.1039/d2ra07900c

**Published:** 2023-02-06

**Authors:** Guangying Zhou, Xiaomeng Cheng, Jian Yang, Yanyan Zhu, Hongping Li

**Affiliations:** a Green Catalysis Center, College of Chemistry, Zhengzhou University Zhengzhou Henan 450001 China lihongping@zzu.edu.cn zhuyan@zzu.edu.cn; b Beijing National Laboratory for Molecular Sciences, CAS Key Laboratory of Colloid and Interface and Thermodynamics, Institute of Chemistry, Chinese Academy of Sciences Beijing 100190 P. R. China; c School of Chemistry and Chemical Engineering, University of Chinese Academy of Sciences Beijing 100049 China

## Abstract

In this work, we use density functional theory (DFT) calculated competitive hydrogen bonds and dissipative particle dynamics (DPD) simulated micellar structural information to uncover the CO_2_-expanded liquid (CXL)-aided self-assembled structure and emission mechanisms of the self-assembled fluorescent composites (SAFCs). Herein, the SAFCs are formed through the self assembly between diblock copolymer polystyrene-*b*-poly(4-vinylpyridine) (PS-*b*-P4VP) blend and the dye molecule 4-(9-(2-(4-hydroxyphenyl)ethynyl)-7,10-diphenylfluoranthen-8-yl)phenol (4) in CO_2_-expanded toluene at 313.2 K and varied pressures. Firstly, from DPD simulation, we have demonstrated that the addition of CO_2_ to toluene favors both the expansion of the solvophobic P4VP phase and contraction of solvophilic PS chains, which facilitates the continuous morphological transitions of SAFCs from spherical micelles (3.0 MPa) through wormlike plus spherical micelles (4.0–4.8 MPa) to large vesicles (6.0–6.5 MPa) with pressure rise. Secondly, the DFT calculated bonding energies and IR spectra of the competitive hydrogen bonds help us to clarify the major type of hydrogen bonds determining the fluorescence (FL) performance of the SAFCs. Furthermore, we have revealed the SAFC emission mechanism *via* the pressure-tunable changes in the aggregation degrees and amount of hydrogen bonds involving 4 and P4VP chains. This work provides a good understanding for the morphology-property control of the self-assembled polymer composites in both microscopic and mesoscopic scales.

## Abstract 2

Computational and experimental approaches on the CXL-aided self-assembled morphology and emission mechanisms of AIE and H-bond featured fluorescent composites.

## Graphical abstract

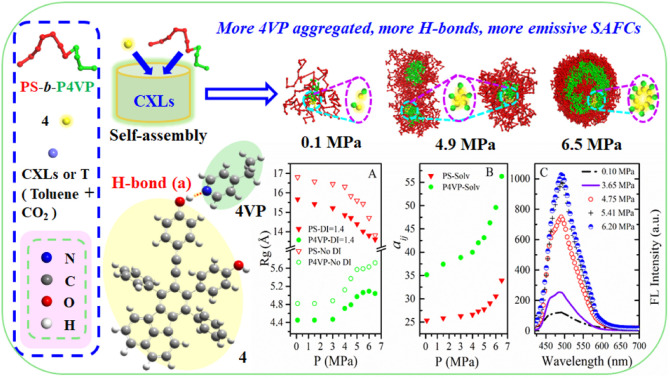

## 1. Introduction

The self-assembly behaviors of amphiphilic block copolymers (ABCs) have received extensive attention because they can generate specific structures *via* spontaneous aggregation based on non-covalent-bond interactions, such as electrostatic interaction,^1,2^ hydrogen bonds (H-bonds),^3,4^ π–π stacking and so on.^5^ Particularly, hydrogen bonds play an attractive role in the progress of self-assembly owing to the stability and dynamic reversibility.^6,7^ So far, ABCs can self-assemble in bulk or solution to form spheres,^8^ rods,^9^ worm-like clusters,^10^ vesicles^11^ and other micellar structures^12,13^ which have many potential applications in drug delivery,^14^ bioimaging,^15^ porous materials template,^16^ and so on.^16,17^ In addition to external factors (temperature and pH), the nature of the self-assembly behaviors depends primarily on the Flory–Huggins parameter (*χ*) of each block with the solvent, and block length, volume fraction, the dispersity index (DI) of polymer, as well as selective solvent^18–20^ and hydrogen bonds.^21,22^ Poly(styrene-*block*-4-vinylpyridine) (PS-*b*-P4VP) is among the most studied block copolymers due to the versatility of the P4VP block,^23^ especially, the P4VP block can act as a hydrogen (H)-bond proton acceptor so that PS-*b*-P4VP becomes an popular amphiphilic block copolymer in solution state self-assembly. Extensive work has been done on the self-assembly of PS-*b*-P4VP with small molecule through H-bonding, ionic interaction, and halogen bonding to form functional materials that have a morphological change with the volume fraction of P4VP block as well as owing to solvent vapor annealing.^23^ Some researchers have reported the aggregation behavior of P4VP block with other molecules *via* hydrogen bonding with the pyridine nitrogen of 4VP.^3,23^ Kuo and co-workers investigated diblock copolymer/homopolymer blends of poly(methyl methacrylate-*b*-4-vinylpyridine)/poly vinylphenol (PMMA-*b*-P4VP/PVPh), where PVPh acted as H-bond donor and PMMA and P4VP as H-bond acceptors. Based on the different H-bonding strength between the binary pairs of PVPh/P4VP and PVPh/PMMA, short range ordered self-assembled structures were formed for pure PMMA-*b*-P4VP copolymer and for its blends with PVPh at lower concentrations, whereas miscible disordered structures were found at higher PVPh concentrations because the OH units of PVPh could both interact with P4VP and PMMA segments.^3^ Roy and co-workers employed a ditopic probe PBI–PDP to investigate the molecular level self-assembly of PS-*b*-P4VP in THF, and a series of complexes were prepared between PS-*b*-P4VP with varying P4VP fractions and PBI–PDP. Detailed information of the assemblies structure, the interaction as well as hydrogen bonds between the probe molecule and copolymer were proved by NMR and FTIR spectra. And the insights could have important implications in tuning the block copolymer micellar structures to suit various application requirements.^23^

Although many experimental studies have been done,^24,25^ the difficulties in characterization limited the fine understanding of the structure–property control of the self-assembled fluorescent composites (SAFCs). Yet, thoroughly understanding the underlying factors that determines the self-assembled structures is essential to help design new materials and nano-devices. In comparison with experimental endeavors, theoretical calculations and simulations have emerged as essential tools to reveal the microscopic message of micelles and provide more detailed micellar structures at a molecular level. Through extensive simulations, the factors influencing the self-assembly can be deeply understood. Recently, many works have been devoted to examining the self-assembly of copolymer by dissipative particle dynamics (DPD) method.^26–30^ Yang and co-workers employed DPD simulations to investigate the mesoscopic behavior of reduction-responsive doxorubicin (DOX) drug-loaded amphiphilic polymeric micelles of PCL-SS-PPEGMA, and the self assembly behavior, formation of DOX-loaded micelles, as well as the DOX reduction-responsive release process were simulated. The simulated results were in good agreement with the experimental data, proving that the DPD method can provide a practical mesoscopic approach for the reduction-responsive polymer micelles.^26^ Li group has also revealed the self-assembly mechanism of the self-assembled aggregates (SAAs) of polystyrene-*b*-poly (2-vinylpyridine) (PS-*b*-P2VP) in a green media through DPD simulation,^30^ and found that the CO_2_-expanded liquids (CXLs) pressure could effectively regulate the structures of SAAs. DPD simulation could help us better understand the morphology control of the self-assembled polymer composites in CXLs, as well as the self-assembly mechanism in a mesoscopic scale, and is very helpful for designing desired self-assembled structures and widening their further applications. Besides, the influence of hydrogen bond on self-assembly behavior was also studied by density functional theory (DFT) method.^31,32^ Zhang and coworkers investigated the self-assembly mechanism of PS-*b*-P4VP and poly(4,4′-oxydiphenylenepyromellitamic acid) (POAA) blends from both microscopic and mesoscopic perspectives by combining DFT and DPD method. The Becke-three Lee–Yang–Parr (B3LYP) method was used to obtain the geometric structures and bonding energies for the possible hydrogen bonds while the morphologies of different blends were studied by DPD simulation. The hydrogen bonds between P4VP and POAA chains, and among POAA chains were confirmed by DFT calculations, and the competitive hydrogen bonds involving POAA controlled the morphology evolution from lamella to sphere.^31^

**Fig. 1:**
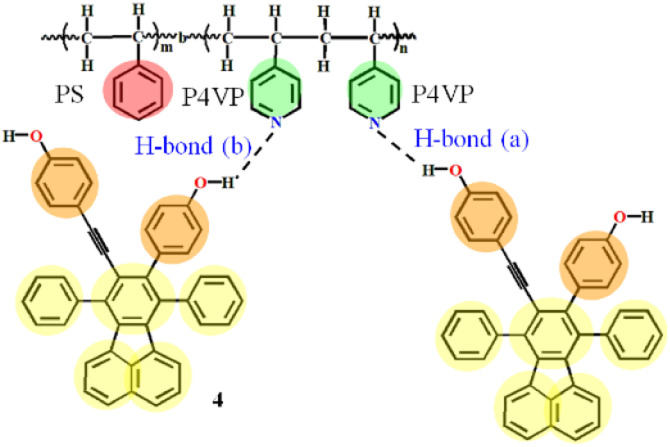
Hydrogen bonding between PS-*b*-P4VP and 4 (dashed lines represent the hydrogen bonding). H-bond (a) stands for the hydrogen bonds between ethynylphenolic OH group of 4 and pyridyl nitrogen atom of P4VP segments, and H-bond (b) between phenolic OH of 4 and nitrogen atom of P4VP.

While the majority of these studies have been taken in conventional solution, very few works are available where the complexes of PS-*b*-P4VP with small molecules are studied in a green media, the CO_2_ expanded liquids (CXLs). As we know, CXLs have outstanding advantages in materials processing compared with conventional solvents due to the desired pressure tunable solvating ability,^33^ which can alleviate the environmental burden of a given process through substantial replacement of organic solvents with eco-friendly compressed CO_2_. CXLs processes can be carried out within mild pressure range, and our group has systematically carried out polymer processes using CXLs to pressure control the morphologies of crystalline polymers,^34^ as well as pressure regulate the structures and emission intensity of the SAFCs of PS-*b*-P4VP with dye molecules 4 or DR1.^24,25^ Particularly, the dye molecule 4 with partially propeller-like moieties and phenolic OH groups can be used as an aggregation-induced emission (AIE)^24,35,36^ and potential H-bond^24,36^ module to further label the self-assembled micellar aggregates and serve as an attractive building block for AIE-active polymer probes. In the work involving the self assembly of P4VP-*b*-PS with 4 in CXLs,^24^ there existed two types of hydrogen bonds, where H-bond (a) and H-bond (b) represented the hydrogen bonds between the pyridyl nitrogen atoms of P4VP blocks and the ethynylphenolic or the phenolic OH group of 4 (Fig. 1). We found that the structures and emission behaviors of SAFCs were strongly pressure dependent, and the emission of SAFCs was decided by two major factors of hydrogen bonding and confinement effect. However, we could not clarify whether H-bond (a) or H-bond (b) is the dominant hydrogen bonds for luminescence control only by experimental FTIR data. Meanwhile, the change in solvated state of P4VP chains with increasing CXLs pressure might influence the number of hydrogen bonds of P4VP with 4. Yet, the experimental TEM images could only provide the overall solvated state of SAFCs clusters instead of the individually solvated information of P4VP segments at molecular level. Besides, it was difficult to distinguish the dominant morphology of SAFCs under a given pressure due to the relatively large experimental DI value (DI = 1.4) of PS-*b*-P4VP,^24^ which generally resulted in multiple SAFCs structures coexisted at a given pressure.

**Fig. 2:**
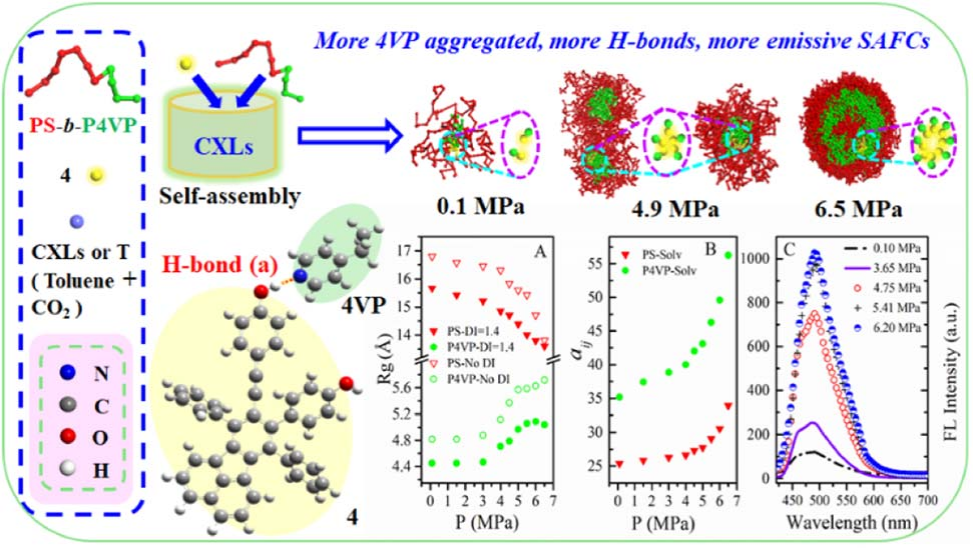
The DPD simulated structural information and experimental FL data of copolymer blends PS_240_-*b*-P4VP_40_/4 in CO_2_–toluene at 313.2 K and varied pressures.

So in this contribution, as schematically shown in Fig. 2, DPD simulations are performed to obtain the structure-governing information of SAFCs such as the CXLs pressure-responsive radius of gyration of P4VP chains (*R*_g,P4VP_), and the change of repulsive (*a*_*ij*_) or Flory–Huggins parameters (*χ*_*ij*_) of each block with CXLs. Meanwhile, the copolymer DI is considered in order to find the major self-assembled morphology at certain pressures. Moreover, DFT calculations are employed to help distinguish the preferential type of hydrogen bonds affecting the luminescence performance of SAFCs. The DFT coupled with DPD approaches to the self-assembly of PS-*b*-P4VP blend with 4 in CO_2_-expanded toluene in this work could help find the predominant factors affecting the morphology and emission control mechanism of SAFCs from micro/mesoscopic scale. The insights will have important influence in tuning the functional polymer micellar structures to suit various applications, and the unique approaches offer a significant and so far missing insight into the self-assembly behaviors in gas expanded liquids.

## 2. Calculation methods and experimental

In this section, DPD and DFT methods are used to investigate the system in different scales. In Section 2.1, the DPD simulation is introduced to obtain the morphological information in mesoscopic dimension, whereas in Section 2.2, the quantum-chemical method of M06-2X is performed to study the hydrogen bonding interactions in microscopic scale. In Section 2.3, the experimental information is briefly described.

### 2.1 DPD simulations

In a mesoscale DPD simulation method,^37,38^ a series of soft beads representing groups of atoms or fluids interact with each other, and all the DPD beads follow Newton's equation of motion.^39^ DPD is a coarse-grained (CG) simulation method suitable for the study of the self assembly process of amphiphilic copolymers,^40^ and the related DPD theory is described in the ESI.†

#### 2.1.1 The coarse-grained (CG) models

**Fig. 3:**
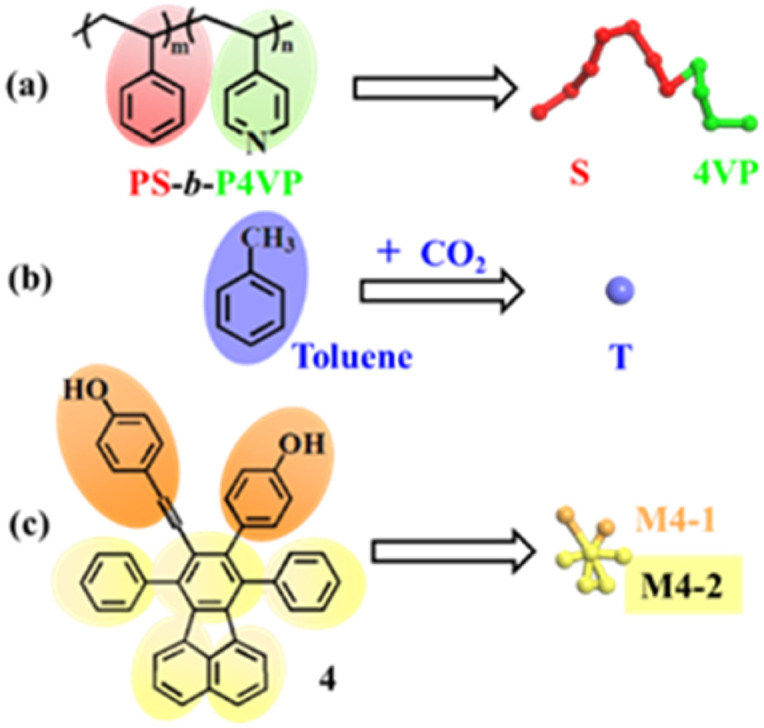
Coarse-grained models of the system. (a) PS-*b*-P4VP; (b) CO_2_–toluene; and (c) 4. Color scheme: S, red; 4VP, green; solvent beads, violet; M4-1, orange; M4-2, yellow.

In this work, we simulate the self-assembly behavior of copolymer PS_240_-*b*-P4VP_40_ blends with 4 in CO_2_-expanded toluene at varied pressures and 313.2 K. A schematic diagram of coarse-grained (CG) models is shown in Fig. 3. Herein, beads S and 4VP denote the repeating units of PS and P4VP of copolymer PS-*b*-P4VP, while bead T stands for the solvent of CO_2_-expanded toluene. Molecule 4 is represented by two types of beads, with beads M4-1 and M4-2 representing the unit of phenol and benzene, respectively.

#### 2.1.2 DPD simulation settings

In this work, the DPD simulations are carried out in a box of size 30 × 30 × 30 (*r*_c_^3^), containing about 8.0 × 10^4^ DPD beads with the density of beads *ρ* = 3 and with periodic boundary conditions under the NVT ensemble. The average mass, radius of each bead and the average volume of beads (*V*_bead_) are 91 amu, 3.39 Å and 163 Å^3^, respectively. The cut-off radius *r*_c_ is the length unit of DPD simulation, being calculated from the formula of *r*_c_ = (3 × *V*_bead_)^1/3^ = 7.88 Å. Herein, the concentrations of copolymer and 4 are larger than those in the experimental study^24^ to ensure a reasonable amount of copolymer/4 interactions.^41^ To match the experimental molar ratio (*R*_M_) of 4VP to 4,^24^ the respective mass ratio of copolymer blend/solvent/4 is set to be 1 : 8.998 : 0.002 with *R*_M_ = 690. The copolymer blend PS_240_-*b*-P4VP_40_ with DI = 1.4 is composed of PS_240_-*b*-P4VP_40_ (mass fraction of 75%), PS_360_-*b*-P4VP_60_ (mass fraction 10%) and PS_120_-*b*-P4VP_20_ (mass fraction 15%). The simulations are performed at temperature *T* = 313.2 K. At least 2.0 × 10^5^ DPD steps (with the time step *δ*_*t*_ = 0.05) are performed for equilibration, and the spring constant is selected as 4.0 according to Groot and Warren.^39^ Besides, the dissipative force coefficient *σ* is set to 4.5, and the random force parameter *ζ* is chosen as 3.^42^ All the DPD simulations are performed using the Materials Studio 2017 from the company BIOVIA.

The detailed procedures for calculating the solubility parameters (*δ*), interaction parameters involving the repulsion parameters (*a*_*ij*_) and Flory–Huggins parameters (*χ*_*ij*_) between pairwise beads can be found in the ESI.† The repulsion parameters between pairwise beads are tabulated in Table 1. Table S1 (ESI†) lists the characteristic ratios and the number of DPD beads of the copolymer PS-*b*-P4VP blend, 4 and solvent CO_2_–toluene. The solubility parameters of solvent beads, the beads of 4 and PS-*b*-P4VP are listed in Table S2 (ESI†), and the Flory–Huggins parameters between pairwise beads are given in Table S3 (ESI†).

**Table 1:** Repulsive parameters *a*_*ij*_ between beads in CO_2_-expanded toluene at 313 K

	M4-1	M4-2	4VP	S
M4-1	25.00			
M4-2	53.95	25.00		
4VP	42.01	26.58	25.00	
S	70.24	26.81	31.77	25.00
T (0.1 MPa)	78.51	28.74	35.18	25.35
T (1.5 MPa)	83.50	30.14	37.42	25.85
T (3.0 MPa)	86.60	31.09	38.87	26.26
T (4.0 MPa)	88.89	31.83	39.97	26.61
T (4.5 MPa)	93.04	33.23	42.01	27.32
T (5.0 MPa)	95.26	34.01	43.13	27.74
T (5.5 MPa)	101.35	36.27	46.29	29.05
T (6.0 MPa)	107.49	38.70	49.58	30.55
T (6.5 MPa)	119.41	43.80	56.27	33.94

### 2.2 Calculations on hydrogen bonding information

The function of M06-2X is frequently used in the study of non-covalent interactions such as hydrogen bonds, and is regarded as one-of the best functional systems with hydrogen bonds.^43,44^ In this work, the equilibrium geometries, interaction energies and IR spectra are calculated by M06-2X method using 6-311++G(d,p) basis set. The interaction energies of complexes are corrected both for basis set superposition error (BSSE) by the Boys–Bernardi full counterpoise method^45^ and for zero-point vibrational energy (ZPE). The frequency analyses have been performed and all the optimized structures have no imaginary frequency, which indicate that the optimized geometries are indeed in the minimum point. As we know, the vibrational frequencies from quantum-chemical calculations are usually larger than their experimental counterparts. Therefore, the calculated frequencies are scaled by 0.972 (ref. 46) to compare with the experimental values. All the calculations above are carried out with the Gaussian 09 program.^47^

### 2.3 Experimental

The general experimental information, materials, the preparation of copolymer PS-*b*-P4VP (*M*_n,PS-*b*-P4VP_ = 29 900 g mol^−1^, *M*_n, P4VP_ = 4300 g mol^−1^) and the self-assembled fluorescent composites (SAFCs) in CO_2_-expanded toluene at 313.15 K and varied pressure were described in the ESI.† The detailed polymerization reaction conditions, the number-average molecular weight (*M*_n_) and dispersity index (*M*_w_/*M*_n_) of PS-*b*-P4VP and P4VP determined by GPC, as well as the block length ratio of PS/P4VP by ^1^H NMR are tabulated in Table S4 (ESI†).

## 3. Results and discussion

Firstly, the CXLs-assisted morphological changes for the PS-*b*-P4VP blend/4 are investigated using DPD simulation. The mechanism of the self-assembly in CXLs is studied, with focus on the influence of CXLs pressure on the self-assembly behavior of SAFCs formed between PS-*b*-P4VP blend and 4 in CO_2_-expanded toluene within pressure range 0.10–6.50 MPa at 313.2 K. The copolymer blend PS_240_-*b*-P4VP_40_ with DI = 1.4 is composed of PS_240_-*b*-P4VP_40_ (mass fraction of 75%), PS_360_-*b*-P4VP_60_ (mass fraction 10%) and PS_120_-*b*-P4VP_20_ (mass fraction 15%), and the molar ratio *R*_M_ of 4VP/4 is set to be 690. Secondly, competitive hydrogen bonding interactions between P4VP segments and 4 are investigated through the geometric structures, bonding energies and IR spectra. Next, the emission mechanism for the CXLs-aided SAFCs of PS-*b*-P4VP blend with 4 involving competitive hydrogen bonds is revealed.

### 3.1 Mesoscopic morphology from DPD simulations

#### 3.1.1 The sequential snapshots of the micelle formation of PS_240_-*b*-P4VP_40_ blend/4 in CO_2_-expanded toluene

**Fig. 4:**
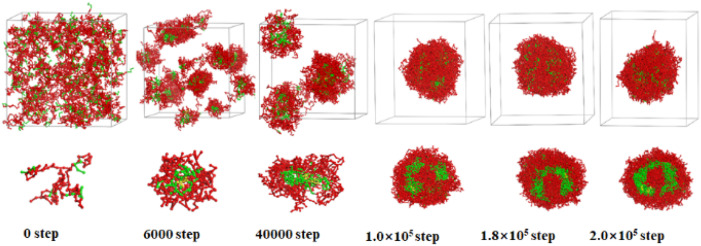
Sequential snapshots of the micelle formation of copolymer blends PS_240_-*b*-P4VP_40_/4 at different simulation time in CO_2_-expanded toluene at 313.2 K and 6.0 MPa. Copolymer blends PS_240_-*b*-P4VP_40_ with DI = 1.4 (mass fraction of 75% PS_240_-*b*-P4VP_40_ + 10% PS_360_-*b*-P4VP_60_ + 15% PS_120_-*b*-P4VP_20_). The images below the box are the cross-section views. The color scheme is the same as in Fig. 3. Solvent beads are hidden.

DPD simulations are performed during the simulation step range 0–2.0 × 10^5^. As an example, the selected morphology evolution of the SAFCs of PS_240_-*b*-P4VP_40_ blend/4 as a function of simulation time in CO_2_-expanded toluene at 313.2 K and 6.0 MPa with *R*_M_ = 690 is presented in Fig. 4. At the beginning (0 DPD step), all components are added into the simulation box randomly, which copolymer blend are dispersed in the solvent, exhibiting dispersed states at 0 step. As the simulation proceeds, the PS-*b*-P4VP blend/4 gradually aggregates into spherical micelles with P4VP cores and PS shells at – 6000 step. Then spherical micelles subsequently merge with the neighboring ones into short wormlike micelles (contour length of rods 112.0 Å) at 4.0 × 10^4^ step. Next, the short wormlike micelles encapsulate solvent and bend its shape to vesicle-like micelles filled with 50% P4VP walls at 1.0 × 10^5^ step, as shown in the cross sections of the micelles (Fig. 4). Finally, with more solvent encapsulated by P4VP segments, a sphere-like vesicle is formed with PS corona and 80% P4VP-filled walls at 1.8 × 10^5^ step, and then a spherical vesicle is formed with 90% P4VP-filled walls at 2.0 × 10^5^ step. The process of vesicle formation in this work is consistent with the mechanism II involving vesicle formation mentioned by Chen *et al.*^48^ At the 2.0 × 10^5^ step, the morphology of the vesicle-like micelles is not further changed with more DPD simulation steps, which indicates that 2.0 × 10^5^ steps (∼48 ns) are sufficient for the system to obtain the equilibrated micellar structures.

#### 3.1.2 The CXLs pressure-tunable morphological evolution of the SAFCs

**Fig. 5:**
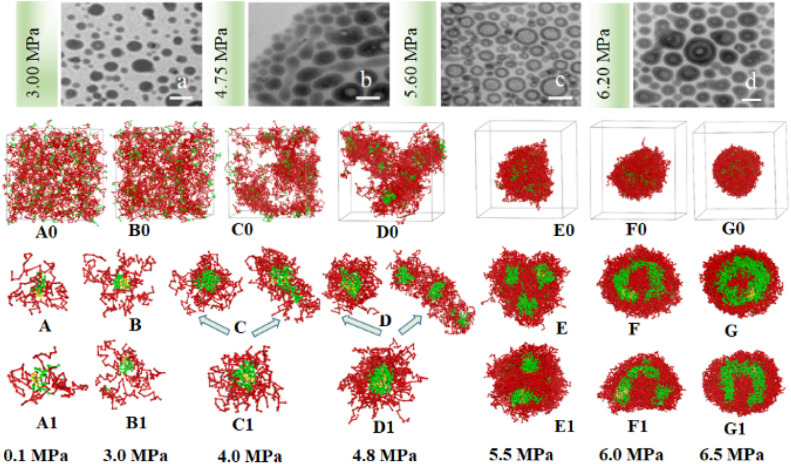
The experimental and simulated SAFCs morphologies of copolymer blends PS_240_-*b*-P4VP_40_/4 in CO_2_-toluene at 313.2 K and varied pressures. (a–d) Experimental TEM images at 3.00, 4.75, 5.60 and 6.20 MPa; DPD simulated structures involving copolymer DI (A0–G0) and (A–G, cross-section views), without copolymer DI (A1–G1, cross-section views). Copolymer blends PS_240_-*b*-P4VP_40_ with DI = 1.4 (mass fraction of 75% PS_240_-*b*-P4VP_40_ + 10% PS_360_-*b*-P4VP_60_ + 15% PS_120_-*b*-P4VP_20_). TEM scale bar at 100 nm. The color scheme is the same as in Fig. 3. Solvent beads are hidden.

From the experimental TEM images shown in Fig. 5a–d, it is found that multiple SAFCs structures are coexisted at certain pressures owing to the relatively large copolymer DI of 1.4. It is vital to determine the major SAFCs morphology at a given pressure to explore the structure dependent emission mechanism of the SAFCs. Therefore, the copolymer DI is introduced to DPD simulation to help find the dominant self-assembled morphology at given pressures. The experimental and DPD simulated SAFCs morphologies of copolymer blends PS_240_-*b*-P4VP_40_/4 in CO_2_-expanded toluene at 313.2 K and varied pressures are shown in Fig. 5. When copolymer DI is not considered, the DPD simulated structures of SAFCs (Fig. 5A1–G1) change from dispersed states (0.1 MPa) through critical – micelles (3.0 MPa) and spheres with loose-packing PS-shells (4.0 MPa), to larger-sized spheres with dense-packing PS-shells (4.8 MPa), and then to vesicle-like micelles filled with 25% P4VP-walls (5.5 MPa) and non-spherical vesicle-like micelles (6.0 MPa) with 60% P4VP-packed walls, and finally to sphere-like vesicles encircled with 80% P4VP-walls at 6.5 MPa. However, as copolymer DI of 1.4 is introduced, the SAFCs morphologies (Fig. 5A–G) transform from slightly aggregated clusters (0.1 MPa, Fig. 5A) through spheres with loose-packing PS-shells (3.0 MPa) to major P4VP-cored spheres plus minor short rods (rod length of 115.0 Å) with more compact P4VP-cores (4.0 MPa), and then to fewer spheres plus dominant longer rods (rod length of 201.0 Å) with loose-linked P4VP-cores at 4.8 MPa (Fig. 5D). Subsequently, the SAFCs structures exhibit vesicle-like micelles merged from fused spheres with 40% P4VP-filled walls at 5.5 MPa (Fig. 5E), and then change to spherical vesicles full of 90% P4VP-walls with thinner P4VP-walls at 6.0 MPa, and finally to perfect spherical vesicles with thicker P4VP-walls at 6.5 MPa (Fig. 5G). In contrast, the experimental conformations of SAFCs micelles transit from spheres (3.00 MPa, Fig. 5a) to spheres plus rods (4.75 MPa), then to dominant vesicles with thinner P4VP-walls (5.60 MPa), and lastly to major vesicles with thicker P4VP-walls at 6.20 MPa (Fig. 5d) with increasing CXLs pressure. So firstly, both experimental TEM images – (Fig. 5a–d) and DPD simulated structures (Fig. 5A1–G1 and A–G) clearly show that the SAFCs morphology control can be realized by facile CXLs pressure tuning. Secondly, by comparing the simulated results (Fig. 5A1–G1 and A–G) with the experimental TEM images (Fig. 5a–d), we find that the simulation without copolymer DI could only output onefold structure rather than multiple morphologies at comparable pressures such as 4.0 and 4.8 MPa. Whereas, the simulation involving copolymer DI (Fig. 5B–G) is able to generate more matched SAFCs structures with the experimental ones (Fig. 5a–d), which further indicates that the introduction of copolymer DI to DPD simulation could be a desired approach to help reveal the self-assembly mechanism of copolymer blends PS-*b*-P4VP/4 in CO_2_-expanded toluene.

**Fig. 6:**
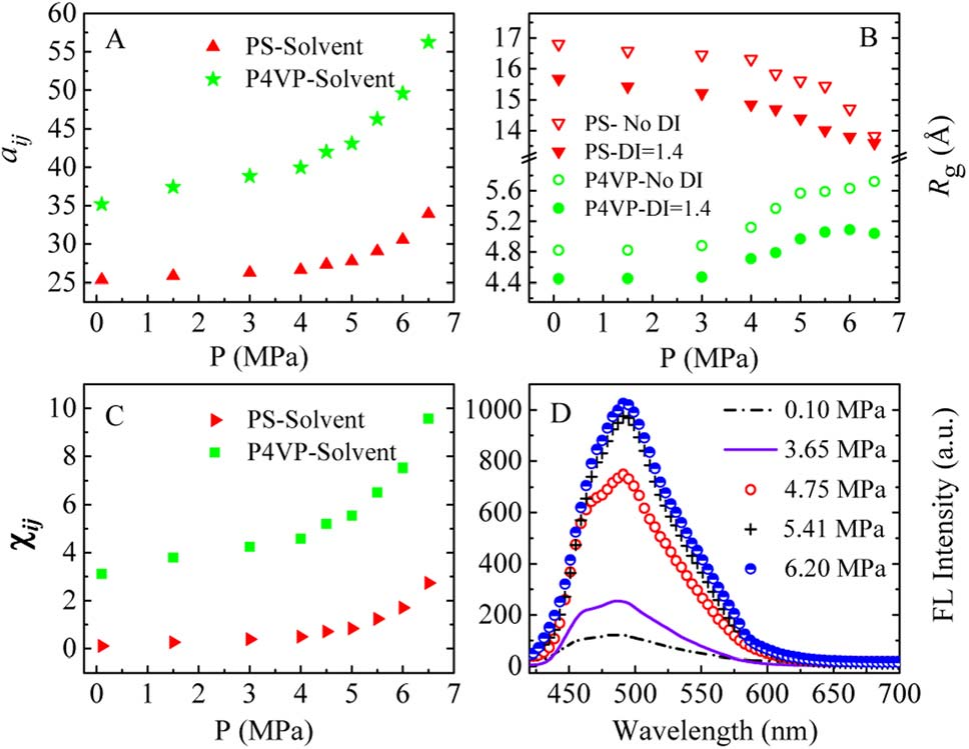
The simulated (A–C) and experimental (D) pressure dependent data in CO_2_–toluene at 313.2 K. (A) Repulsive parameters *a*_*ij*_ of PS–solvent and P4VP–solvent bead pairs; (B) radius of gyration (*R*_g_) of different blocks of copolymer blends PS_240_-*b*-P4VP_40_ with and without considering copolymer DI; (C) Flory–Huggins parameters *χ*_*ij*_ of PS–solvent and P4VP–solvent bead pairs; (D) experimental FL spectra of the SAFCs of PS-*b*-P4VP/4 in CO_2_-expanded toluene (313.2 K/varied pressure for 72 h). Copolymer blends PS_240_-*b*-P4VP_40_ with DI = 1.4 (mass fraction of 75% PS_240_-*b*-P4VP_40_ + 10% PS_360_-*b*-P4VP_60_ + 15% PS_120_-*b*-P4VP_20_), “Solvent” denotes the solvent bead of CO_2_–toluene.

We speculate that addition of CO_2_ to toluene might alter the solvophobic/solvophilic balance of copolymer segments and influence the packing geometry for the PS–P4VP interface, and help realize the morphology control *via* facile pressure tune of CXLs. Therefore, the SAFCs structures are investigated from micro/meso-scale, focusing on how pressure affects the structure-determining factors such as solubility parameters (Table S2 in ESI†), Flory–Huggins parameters (Table S3 (ESI†) and Fig. 6C), repulsive parameters (Table 1 and Fig. 6A), as well as the radius of gyration of – polymer segments (Fig. 6B). First, from the repulsive parameters of unlike-bead pairs shown in Table 1, the *a*_*ij*_ of 4VP–M4-1 pair (*a*_4VP–M4-1_ = 42.01) is smaller than that of S–M4-1 pair (*a*_S–M4-1_ = 70.24), indicating that molecule 4 prefers to locate at the site more close to P4VP segments in the micelles. Meanwhile, within pressure range 0.10–6.50 MPa, the repulsive parameters (Fig. 6A) of P4VP–solvent beads pair (*a*_P4VP–solv_) are always much larger than those of PS–solvent beads pair (*a*_PS–solv_), suggesting that P4VP-chains are more solvophobic segments and should be located in the inner part of the micelles (*e.g.*, in cores or vesicle wall parts), which matches well with the simulated cross-section structures shown in Fig. 5A–G. Besides, both *a*_P4VP–solv_ and *a*_PS–solv_ increase with CXLs pressure rise (Fig. 6A), signifying enhanced clustering degree of polymer segments and overall micellar aggregates in order to decrease the total interfacial free energy and stabilize the micellar system, which agrees well with the pressure-responsive overall simulated structures shown in Fig. 5A0–G0. Second, the changes of radius of gyration (*R*_g_) could reflect the curling degree of polymer segments against varying conditions under constant polymer composition.^49^ In order to reveal the influence of CXLs pressure on SAFCs structure, the *R*_g_ changes of copolymer segments against CXLs pressure are examined, and the results are shown in Fig. 6B. The CXLs pressure increases with the addition of more CO_2_ to toluene, and as pressure increases from 0.1 to 6.0 MPa, the radius of gyration of PS segments (*R*_g_,_PS_) keeps decreasing with pressure rise, together with a *R*_g_,_PS_ reduction of 13.6% and 17.7% for DI = 1.4 and without DI. Whereas the radius of gyration of P4VP chains (*R*_g_,_4VP_) increases with pressure enhancement, along with a *R*_g_,_4VP_ increase of 14.4% and 18.7% for DI = 1.4 and without DI. Generally, a contraction in corona blocks prefers the formation of less curved rod-like or succeeding vesicular micelles with smaller core/corona interface, and more contracted solvophilic chains that stabilize a lower total surface area will promote the micellar morphological shift from spheres through cylinders to vesicles.^50^ That is, the addition of more CO_2_ to toluene favors both the continuous expansion of the solvophobic P4VP phase and contraction of solvophilic PS chains, which facilitates and explains the observed SAFCs morphological evolution from spheres (3.0 MPa) *via* rods (4.0–4.8 MPa) to vesicles (5.5–6.5 MPa) with pressure rise shown in Fig. 5. Third, as shown in Table S2 (ESI†) and Fig. 5A, at 0.1 MPa (free of CO_2_), the dissolution of PS segments occurs because the solubility parameter of PS (*δ*_PS_ = 18.57) is close to that of the solvent (*δ*_toluene_ = 18.04), which results in very small Flory–Huggins parameter *χ*_*ij*_ of PS/toluene based on eqn (S6) in ESI.† Yet, the addition of CO_2_ changes the *χ*_*ij*_ of polymer blocks and the solvent. The respective Flory–Huggins parameter of PS/CO_2_–toluene (*χ*_PS–solv_) and P4VP/CO_2_–toluene (*χ*_P4VP–solv_) increase 24.8- and 3.1-fold as pressure increases from 0.1 to 6.5 MPa (Fig. 6C), which leads to a rise in the total interfacial tension (*γ*), as *γ* is directly proportional to the Flory–Huggins parameter of polymer–solvent, *χ* (*γ* ∼ *χ*^1/2^).^50^ Therefore, the formation of spherical micelles at low pressures (3.0 and 4.0 MPa) might be favored due to a lower total interfacial tension. While as pressure further increases, the transition from rods (4.8 MPa) to vesicles (5.5–6.5 MPa) is a consequence of the further reduced surface area of the rod-like micelles originating from further contraction of the PS corona upon increasing pressure. In other words, the decrease in PS corona volume as well as increase in total interfacial tension both promote morphology toward rod-like and subsequent vesicular micelles with increasing CXLs pressure. The observed micellar geometrical shifts from spheres to rods and then to vesicles (Fig. 5A–G) against pressure satisfy the criteria to reduce the total interfacial free energy and stabilize the micellar system.

Finally, we can also employ packing geometry model to explain the observed SAFCs structures against pressure (Fig. 5a–d and A–G). For AB diblock copolymers, packing geometry defined by packing parameter *p* can be formulated as *p* = *V*_B_/*a*_0_*l*_B_, in which *V*_B_ and *l*_B_ stand for the volume and length of the solvophobic block, respectively, and *a*_0_ represents the effective contact area of the solvophilic segment.^51^ According to packing geometry model, the self-assembled structures of amphiphilic copolymers usually vary from spherical micelles (*p* ≤ 1/3) to cylinders (1/3 ≤ *p* ≤ 1/2) and then to vesicles (1/2 ≤ *p* ≤ 1) and inverted structures (*p* > 1) as *p* increases.^52^ Herein, the enhanced *χ*_PS–solvent_ with increasing pressure (Fig. 6C) suggests the worsening miscibility of PS segments with solvent, which brings about contraction of PS corona and a reduction in the contact area *a*_0_ between PS and CO_2_–toluene. Therefore, the increase in *R*_g_,_4VP_ and *χ*_PS–solvent_ with increasing pressure results in a simultaneous volume expansion of solvophobic P4VP chains (*V*_B_) and a drop in the contact area (*a*_0_) of PS segment, which leads to a rise in packing parameter (*p* = *V*_B_/*a*_0_*l*_B_) with increasing pressure. Hence, the observed SAFCs morphology evolution from spheres (3.0 MPa, Fig. 5a and B) to rods plus spheres (4.0–4.8 MPa, Fig. 5b and D) and then to vesicles (6.0–6.5 MPa, Fig. 5d and F, G) together with enhanced packing parameter conforms to the packing geometry model very well.

In brief, these results demonstrate convincingly from micro/meso-scale level that CXLs pressure has a crucial impact on the morphology control of the SAFCs, and can be utilized to fine tune the self-assembled nanostructures.

### 3.2 H-bonding interactions between P4VP chains and 4 by DFT calculation

In the system of P4VP-*b*-PS and 4 in CO_2_–toluene, H-bond (a) and H-bond (b) represented the hydrogen bonds (H-bonds) between the nitrogen atoms of the pyridine ring of P4VP blocks and the ethynylphenolic or the phenolic OH group of 4, respectively (Fig. 1). Although the H-bonds between P4VP blocks and 4 were confirmed by FTIR data and thought to be one of the major factors to decide the emission performance of the SAFCs,^24^ we still could not clarify whether H-bond (a) or H-bond (b) was the dominant factor for emission control only from experimental study. Thus, in the following section, we will focus on the comparison between H-bond (a) and H-bond (b) through the geometry structures, bonding energies and IR spectra analysis, and try to illustrate the dominant type of H-bonds to modulate the SAFCs emission.

#### 3.2.1 Geometry structures for hydrogen bonding

In the blends of PS-*b*-P4VP/4, it is possible to form inter-molecular hydrogen bonds between OH group (both proton donors and acceptors) of 4 and nitrogen in pyridine ring of P4VP (proton acceptors), as well as between OH groups of 4 unit. Yet, we are not sure whether intra-molecular hydrogen bonds could be formed between ethynylphenolic OH group and phenolic OH group of 4 moiety. Fig. S1 (ESI†) shows the optimized stable structures for H-bonds (a) and H-bonds (b) in the SAFCs using M06-2X method, as well as the optimized distances between the potential O and H atoms in 4 moiety. Here the intra-molecular H-bonds in 4 molecules are unable to form because the optimized distance between the potential O and H atoms is 8.38 and 9.29 Å, respectively, exceeding the reasonable hydrogen bond length. Besides, the molar ratio of 4VP to 4 is set to be 690 in this work, which means very low concentration of 4 in the system. Hence the inter-molecular H-bonds between 4 molecules can be neglected because it is difficult to form such H-bonds, and the relative amount of such H-bonds is too low to have any important influence on the SAFCs emission.

Table S5 (ESI†) lists the DFT-calculated distances and angles involved in the hydrogen bonding between 4 and P4VP chains. The distances of hydrogen bond obtained from calculations are in good accordance with the reference value (Table S5 (ESI†)),^53^ indicating the accuracy of the DFT calculations. Bond length has a close relation with the bond strength, for similar chemical environment, the shorter of the bond the stronger of the bond strength.^31^ The respective hydrogen-bonding length *R*_O–H⋯N_ for complexes involving H-bonds (a) and H-bonds (b) is 1.744 and 1.763, which indicates the hydrogen bonding between ethynylphenolic OH group (O–H_EP_) of 4 and nitrogen atom of P4VP segments, the H-bonds (a), is stronger than H-bonds (b) between phenolic OH (O–H_P_) of 4 and nitrogen atom of P4VP chains, as shown in Table S5 (ESI†). Moreover, the bond angles (*θ*(O–H⋯N)) for H-bonds (a) and H-bonds (b) are 175.5 and 175.0°, respectively, which satisfies the criteria for hydrogen bonds,^31^ and further suggests stronger H-bonds (a) than H-bonds (b) due to the former one being more close to 180° than the latter one. Meanwhile, owing to the formation of H-bonds between P4VP and 4, the angle *θ*(C–N–C) in pyridine ring of 4VP increases from 116.6 to 117.9°, together with a rise from 109.5 to 110.9° for the angle *θ*(C–O–H) in phenol unit of 4. That is, the widening of angles involved in the hydrogen bonding also favors the formation of H-bonds between P4VP and 4.

#### 3.2.2 The interaction energy of hydrogen bonding

In order to make clear the relationship between stability and hydrogen bonding, the relative bonding energies Δ*E* and Δ*E*_BSSE+ZPE_ – (including the BSSE and ZPE corrections) for hydrogen bonding of the SAFCs are calculated using M06-2X method (Table 2). The respective bonding energy Δ*E*_BSSE+ZPE_ for SAFCs involving H-bonds (a) and H-bonds (b) is 39.14 kJ mol^−1^ and 36.91 kJ mol^−1^. Herein, the bonding energy order agrees well with the bond-length order shown-in Table S5 (ESI†), and from the above orders, we are able to confirm that H-bonds (a) has relative higher bonding energy and stronger hydrogen bonding strength.

**Table 2:** Bonding energies (kJ mol^−1^) for the hydrogen bonding

Structure	Δ*E* (kJ mol^−1^)	ZPE (kJ mol^−1^)	BSSE (kJ mol^−1^)	Δ*E*_BSSE+ZPE_^a^ (kJ mol^−1^)	Δ*E*_BSSE+ZPE_ (ref.) (kJ mol^−1^)
H-bond (a)	−44.90	2.54	3.22	−39.14	−46.97 (ref. 31); −50.30 (ref. 54)
H-bond (b)	−42.94	2.73	3.30	−36.91	

a Corrected by basis set superposition error (BSSE) and zero-point vibrational energy (ZPE).

The energies of hydrogen bond obtained from calculations are close to the reference values shown in Table 2, further showing the accuracy of the DFT calculations. For hydrogen-bonded SAFCs, the BSSE corrections are in the range 7.2–7.7% of the uncorrected Δ*E*, while the proportions of ZPE to the uncorrected Δ*E* are in the range 5.7–6.4%. The BSSE corrections are relatively larger than that of ZPE corrections, and the two corrections are essential for the calculations of hydrogen-bonding energies.

#### 3.2.3 IR spectra and vibrational frequency analysis

**Fig. 7:**
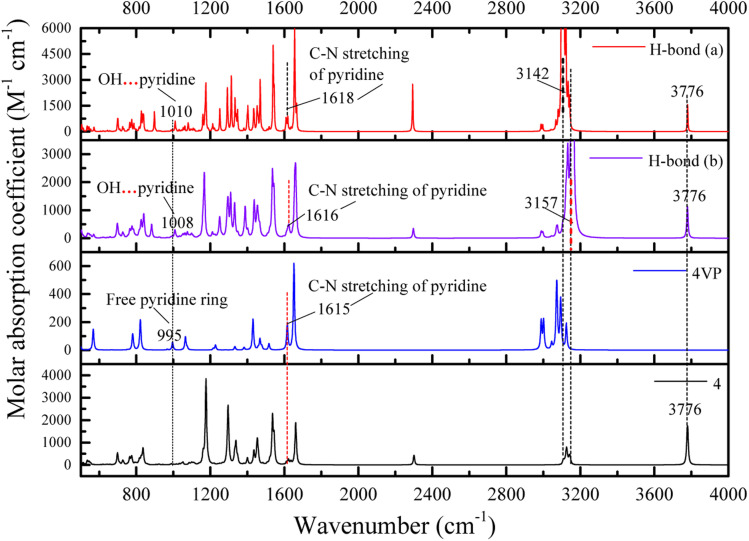
DFT calculated IR spectra for the hydrogen bonding between P4VP and 4 of the SAFCs.

**Fig. 8:**
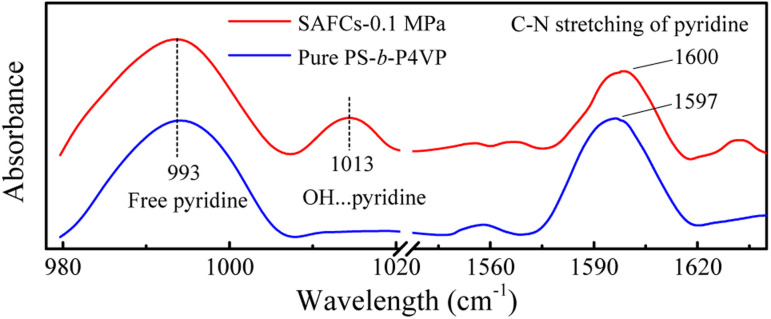
The experimental IR spectra of pure PS-*b*-P4VP and the SAFCs of PS-*b*-P4VP/4 in CO_2_-expanded toluene (313.2 K/0.1 MPa for 72 h).

The frequency difference (Δ*ν*) between the free group and that of the hydrogen-bonded one is a measure of the strength of the interactions.^31^ Herein, IR spectra of different structures related with hydrogen bonding are obtained through frequency calculations, and Fig. 7 shows the calculated IR spectra for the hydrogen bonding between P4VP chains and 4. For free molecule 4, the O–H stretching vibration is at 3776 cm^−1^ (3828 (ref. 55)), while the strong absorption peaks at 3157 cm^−1^ and 3142 cm^−1^ can be assigned to the hydrogen bonding of O–H_P_ (phenolic OH group) and O–H_EP_ (ethynylphenolic OH group) to the aromatic C=N of pyridine ring, respectively. The frequency shifts are about 619 cm^−1^ for O–H_P_ and 634 cm^−1^ (632 (ref. 55)) for O–H_EP_, respectively, which proves the O–H_EP_ hydrogen bond (H-bond (a)) is stronger than the O–H_P_ one (H-bond (b)) for the same proton acceptor P4VP. While for free P4VP, the calculated characteristic bands sensitive to the formation of hydrogen bond are located at 995 cm^−1^ (993 (ref. 24)) for free pyridine ring and 1615 cm^−1^ (1597 (ref. 24)) due to the aromatic C–N stretching. As shown in Fig. 7, the calculated free C–N of pyridine stretching vibrations at 1615 cm^−1^ shifts to higher wavenumbers of 1618 cm^−1^ (H-bond (a)) and 1616 cm^−1^ (H-bond (b)) upon the addition of 4, indicating the formation of hydrogen bonds between OH groups of 4 and the pyridine groups of P4VP block.^24^ Meanwhile, relative to free pyridine ring absorption band at 995 cm^−1^, the new band observed near 1008 cm^−1^ (H-bond (b)) or 1010 cm^−1^ (H-bond (a)) corresponds to the hydrogen-bonded pyridine units.^24^ That is, another proof for the formation of hydrogen bond between 4 and P4VP can be found from the frequency shift (Δ*ν*) of P4VP characteristic bands, sensitive to the formation of hydrogen bond, at 995 cm^−1^ (Δ*ν* = 20,^24^ 15 for H-bond (a) and 13 for H-bond (b)) and 1615 cm^−1^ (Δ*ν* = 3,^24^ 3 for H-bond (a) and 1 for H-bond (b)), and the shift direction in this work is consistent with that in ref. 24 and the experimental IR spectra of the SAFCs shown in Fig. 8. In contrast, the experimental H-bond proof involving P4VP chains can be found from the respective frequency shift, the shift of free pyridine from 993 to 1013 cm^−1^ (SAFCs), and the aromatic C–N stretching vibrations from 1597 to 1600 cm^−1^ (SAFCs) shown in Fig. 8.

The frequency shifts Δ*ν* for functional groups involving hydrogen bonds are shown in Table 3. For proton acceptor C=N from pyridine ring of P4VP, the frequency shift order from different proton donors is: O–H_P_ (phenolic OH group) < O–H_EP_ (ethynylphenolic OH group), indicating the hydrogen bonds for H-bond (a) are relatively stronger, owing to different electro-negativity of O–H_P_ and O–H_EP_ groups. In other words, the hydrogen bonds between P4VP and O–H_EP_ (H-bonds (a)) are stronger than that between P4VP and O–H_P_ (H-bonds (b)). The same conclusions are also obtained from the calculated bond lengths (Table S5 (ESI†)) and bonding energies (Table 2), which supports the competitive advantage of H-bonds (a) over H-bonds (b) in the formation of hydrogen bonds between P4VP and 4.

**Table 3:** IR vibrational frequency (*ν*) and frequency shifts (Δ*ν*) for different functional groups due to hydrogen bonding^a^

Structure	Group	*ν* (free) cm^−1^	*ν* (bonded) cm^−1^	Δ*ν* cm^−1^
H-bond (a)	Pyridine^b^	995 (993^f^)	1010 (1013^f^)	15 (20^f^)
	Pyridine^c^	1615 (1597^f^)	1618 (1600^f^)	3 (3^f^)
	O–H_EP_^d^	3776 (3828^g^)	3142 (3196^g^)	634 (632^g^)
H-bond (b)	Pyridine^b^	995 (993^f^)	1008	13
	Pyridine^c^	1615 (1597^f^)	1616 (1600^f^)	1 (3^f^)
	O–H_P_^e^	3776 (3828^g^)	3157 (3196^g^)	619 (632^g^)

a Data given in parentheses refer to the corresponding values from references.

b Pyridine ring of 4VP.

c Stretching of pyridine group of 4VP.

d Stretching of ethynylphenolic OH group of 4.

e Stretching of phenolic OH group of 4.

f From ref. 24.

g From ref. 55.

The IR spectrum data calculated by DFT method are in good accordance with the experimental IR spectra (Fig. 8) as well as the experimental data.^24^ Moreover, the calculated IR data can help to confirm and distinguish species of hydrogen bonding. The appearance of new bands at the higher frequency side of free pyridine ring absorption offers a proof for the formation of hydrogen bonds. Hence, the dominant type of hydrogen bonds, the H-bond (a), can be easily distinguished from IR data using DFT calculations.

### 3.3 The pressure-tunable emission mechanism of the SAFCs combined with DPD simulation and DFT calculations

The copolymer PS-*b*-P4VP/4 blend could form the SAFCs in CO_2_-expanded toluene *via* the self assembly. From the repulsive parameters of unlike-bead pairs (Table 1), the repulsive parameter *a*_4VP–M4-1_ of 42.01 is much smaller than *a*_PS–M4-1_ of 70.24, which means that molecule 4 prefers to locate at the site closer to P4VP chains in SAFCs micelles. In addition, in Section 3.2, we have confirmed the formation of hydrogen bonds between P4VP blocks and 4 by DFT calculated results (Table 3 and Fig. 7). Consequently, the solvophobic P4VP block should be located in the inner part of the micelles, together with molecule 4 anchored in the same location as P4VP segments through hydrogen bonding with P4VP. As we know, the dye molecule 4 containing both partially propeller-like moieties and phenolic OH group makes itself a suitable aggregation induced emission (AIE) and hydrogen-bonding module, where the propeller-like moieties endow 4 with the AIE feature.^24,35,36^ Therefore, either the hydrogen bonding coupling of 4 with P4VP chains, or the enhanced aggregation degree of P4VP chains around 4, will result in the restricted intramolecular rotation (RIR) of 4 and thus promote its light emission. In other words, the formation of hydrogen bonds between 4 and P4VP chains, and the aggregation of 4 and P4VP chains are two major factors to decide the SAFC emission. Therefore, in the following section, we will reveal the SAFCs emission mechanism *via* the pressure-tunable changes in the aggregation degrees and amount of hydrogen bonds involving 4 and P4VP chains.

The experimental FL spectra of the SAFCs of PS-*b*-P4VP/4 in CO_2_-expanded toluene at 313.2 K and varied pressures are given in Fig. 6D. As shown in Fig. 6D, the emission of SAFCs keeps continuously increased with CXLs pressure rise in the range of 0.10–6.20 MPa. Meanwhile, in Section 3.1, we have demonstrated that the addition of more CO_2_ to toluene favors both the expansion of the solvophobic P4VP phase and contraction of solvophilic PS chains, which facilitates the continuous SAFCs morphological transitions from spherical micelles (3.0 MPa) through wormlike micelles (4.0–4.8 MPa) to large vesicles (6.0–6.5 MPa) with pressure rise (Fig. 5). To further evaluate the pressure tunable number of P4VP chains in particular types of micelles, the structural data of SAFCs micelles (copolymer DI = 1.4) from DPD simulations are tabulated in Table S6,† including the number of copolymer chains in particular types of micelles and typical contour length of the worm-like micelles. From Table S6,† the number of copolymer chains is 28 for spherical micelles at 3.0 MPa, and 97 at 4.0 MPa (containing 44 for spheres plus 53 for short rods), while the number of copolymer chains is 203 at 4.8 MPa (including 62 for spheres plus 141 for longer rods) and then 323 for large vesicle-like micelles (5.5–6.5 MPa). Therefore, from Table S6† and Fig. 5A–G, apparently more amount of P4VP chains is confined in the wall-part of the vesicular micelles at 6.0–6.5 MPa than those in the inner portion of wormlike plus spherical micelles (4.8 and 4.0 MPa) and those in the slightly aggregated clusters at 0.1 MPa, and thus more enhanced aggregation degree of P4VP segments around 4 moiety will lead to stronger restricted intramolecular rotation of 4 molecules, and therefore result in more emissive of SAFCs with increasing pressure, which is in good accordance with the experimental FL variation trend against pressure (Fig. 6D). It indicates that the aggregation effect of 4 arising from the pressure tunable enhanced clustering degree of P4VP blocks is one of the dominant factor to control the emission of SAFCs.

In this work, the molar ratio of 4 to 4VP is as low as 1 : 690, which means the more P4VP chains around 4 in the local micro-environment or micelles, the more amount of hydrogen bonds will be formed between P4VP and 4. As shown in Fig. 6B, the radius of gyration of P4VP chains becomes larger as CXLs pressure increases, which means more extended P4VP chains and thus favors the formation of more hydrogen bonds with 4 with increasing pressure. Simultaneously the interaction parameters *a*_P4VP–solv_ and *χ*_P4VP–solv_ (Fig. 6A and C) both keep increased as CXLs pressure rises, suggesting more enhanced clustering degree of P4VP segments in order to reduce the interfacial free energy of micelles. That is, as CXLs pressure increases, there will be more P4VP chains gathered inside the micelles, and the aggregated degree of P4VP chains becomes more and more enhanced (Fig. 5A–G). Hence, as pressure increases in the range of 0.1–6.0 MPa, larger *R*_*g*,4VP_ accompanied by more amount and more aggregated P4VP chains around 4 inside the SAFCs micelles, will certainly lead to more amount of hydrogen bonds between P4VP and 4, and result in stronger restricted intramolecular rotation of 4, and thus make the SAFCs more and more emissive with increasing pressure, which also agrees well with the pressure dependence of experimental FL change of SAFCs (Fig. 6D).

Moreover, we have demonstrated that H-bond (a) between ethynylphenolic OH group of 4 and P4VP is stronger than H-bond (b) between phenolic OH group of 4 and P4VP based on the calculated bond lengths (Table S5 in ESI†), bonding energies and IR data (Tables 2 and 3 and Fig. 7). Therefore, we could confirm convincingly that the pressure tunable amount of hydrogen bonds is another important factor to decide the SAFCs emission, particularly H-bond (a) is the preferential and dominant type of hydrogen bonds that determines the SAFCs emission.

## 4. Conclusions

We have combined DFT, DPD and experimental approaches to study the self-assembly of PS-*b*-P4VP blend and dye molecule 4 in CO_2_-expanded toluene at 313.2 K within pressure range of 0.10–6.50 MPa. The DFT calculated competitive hydrogen bonding and the DPD simulated micellar structure information help us distinguish the dominant CXLs-aided self-assembled morphology and emission mechanisms of SAFCs from both microscopic and mesoscopic scales. From DPD simulation, we demonstrate that addition of CO_2_ to toluene alters the solvophobic/solvophilic balance of copolymer and influences packing geometry for the PS–P4VP interface, based on the calculated pressure dependence of structure-determining properties such as Flory–Huggins parameters, repulsive parameters, as well as the radius of gyration of polymer segments. The SAFCs emission mechanism is revealed *via* the pressure-tunable changes in the aggregation degrees and amount of hydrogen bonds involving 4 and P4VP blocks of copolymer. Moreover, from the DFT calculated bond lengths, bonding energies and IR spectra of the competitive hydrogen bonds, we have confirmed that H-bond (a) is the dominant type of hydrogen bonds, and the pressure tunable amount of hydrogen bonds is another major factor other than the aggregation degrees of P4VP blocks to control the SAFCs emission. In brief, this work provides a good understanding for the morphology-property control of the self-assembled polymer composites in both microscopic and mesoscopic scales, and the insights will have important influence in tuning the functional polymer micellar structures to suit various applications.

## Author contributions

G. Y. Zhou, X. M. Cheng and H. P. Li proposed the project, designed the simulations/calculations/experiments and wrote the manuscript; G. Y. Zhou performed and analyzed the DPD simulations; X. M. Cheng did the experiments and related data analysis; J. Yang performed and analyzed the DFT calculations; Y. Y. Zhu supervised the DFT calculations; H. P. Li supervised the whole project.

## Conflicts of interest

There are no conflicts of interest to declare.

## Supplementary Material

RA-013-D2RA07900C-s001
