# Supplementary material for: Computational and experimental studies on the micellar morphology and emission mechanisms of AIE and H-bonding fluorescent composites
Source: RSC Adv. 2023 Feb 6;13(7):4612–22. doi: 10.1039/d2ra07900c (PMC9900601; doi:10.1039/d2ra07900c)
Supplement: RA-013-D2RA07900C-s001 [file RA-013-D2RA07900C-s001.pdf]

Electronic Supplementary Material (ESI) for RSC Advances.
This journal is © The Royal Society of Chemistry 2023

Supporting Information

Computational and experimental studies on the micellar morphology and emission mechanisms of an AIE and H-bond featured fluorescent composites

Guangying Zhou[a], Xiaomeng Cheng[b,c] , Jian Yang[a], Yanyan Zhu[a, *], Hongping Li[a, *]

[a] Green Catalysis Center, and College of Chemistry, Zhengzhou University, Zhengzhou, Henan 450001, China

[b] Beijing National Laboratory for Molecular Sciences, CAS Key Laboratory of Colloid and Interface and Thermodynamics, Institute of Chemistry, Chinese Academy of Sciences, Beijing 100190, P.R. China

[c] School of Chemistry and Chemical Engineering, University of Chinese Academy of Sciences, Beijing 100049, China

E-mail: zhuyan@zzu.edu.cn ; lihongping@zzu.edu.cn.

Simulation methods and experimental information

1. DPD simulations

1.1 DPD simulation theory. In a mesoscale DPD simulation method,[1,2] a series of soft beads representing groups of atoms or fluids interact with each other, and all the DPD beads follow Newton's equation of motion.[3] It can simulate the system with coarse-grained (CG) models over longer length and time scales. The total non-bonded force acting on a DPD bead i, $\boldsymbol{f_i}$ is given by a sum of conservative force ($\boldsymbol{F}_{ij}^{\boldsymbol{C}}$), dissipative force ($\boldsymbol{F}_{ij}^{\boldsymbol{D}}$), and random force ($\boldsymbol{F}_{ij}^{\boldsymbol{R}}$). Each $\boldsymbol{f_i}$ is pair wise additive,[3]

$$\boldsymbol{f}_i = \sum_{j \neq i} \left(\boldsymbol{F}_{ij}^{\mathrm{C}} + \boldsymbol{F}_{ij}^{\mathrm{D}} + \boldsymbol{F}_{ij}^{\mathrm{R}} \right) \tag{S1}$$

The sum of the forces acts on all beads within the cutoff radius r_c, beyond which the forces are ignored. And the three forces are described by the following equations:

$$\boldsymbol{F}_{ij}^{C} = \begin{cases} a_{ij}(1-r_{ij})\hat{r}_{ij} & (r_{ij} < 1) \\ 0 & (r_{ij} \geq 1) \end{cases} \tag{S2}$$

$$\boldsymbol{F}_{ij}^{D} = -\frac{\sigma(\omega(r_{ij}))^2}{2kT}(\mathbf{v}_{ij} \cdot \hat{r}_{ij})\hat{r}_{ij} \tag{S3}$$

$$\boldsymbol{F}_{ij}^{R} = \frac{\sigma\omega(r_{ij})\hat{r}_{ij}\zeta}{\sqrt{\delta_t}} \tag{S4}$$

where σ *and* ζ denote the noise strength and a randomly fluctuating variable, and δ_t, k and T *represent* the time step of DPD simulation, Boltzmann constant, and the system temperature. The r-dependent weight function $\omega(r) = 1 - r$ for $r < 1$ or $\omega(r) = 0$ for $r \geq 1$. For two unlike beads i and j, the relationship between repulsion parameter (a_{ij}) and Flory-Huggins parameter (χ_{ij}) was proposed by Groot and Warren as shown in equation (S5),[3]

$$a_{ij} \approx a_{ii} + 3.27\chi_{ij} \quad \text{for } \rho = 3 \tag{S5}$$

where the repulsion parameter between two alike beads a_{ii} is set to be 25.00, and the Flory-Huggins parameter between two unlike beads i and j (χ_{ij}) can be obtained from the solubility parameter (δ) by equation (S6),[4,5]

$$\chi_{ij} = \frac{V_b}{RT}\left(\delta_i - \delta_j\right)^2 \quad \text{(S6)}$$

Where δ_i and δ_j are the solubility parameters of a pair of interacting beads, V_b is the arithmetic average of molar volume of the beads, R is the gas constant, and T the system temperature.

1.2 The coarse-grained (CG) models. The coarse-grained models is shown in Fig. 3 (main text). In this CG scheme, all beads have similar volume and the molecular topology is maintained. The number of DPD beads in each mesoscale molecule is determined by the characteristic ratio (C_n) and the degree of polymerization of the polymer. The DPD chain length (N_{DPD}) is expressed by equation (S7),

$$N_{DPD} = \frac{M_{\mathrm{p}}}{M_{\mathrm{m}} C_{\mathrm{n}}} \quad \text{(S7)}$$

Where M_{p}, M_{m} stand for the molecular weight of polymer and the monomer. The characteristic ratios are obtained from the synthia module of Materials Studio 2017 (BIOVIA). In this work, we simulate the self-assembly behavior of copolymer PS_{240}-*b*-$P4VP_{40}$ blends with 4 in CO_2-expanded toluene at varied pressures and 313.2 K. Table S1 lists the characteristic ratios and the number of DPD beads of the copolymer PS-*b*-P4VP blend, 4 and solvent CO_2-toluene. The DPD chains for PS_{240}-*b*-$P2VP_{40}$, PS_{360}-*b*-$P4VP_{60}$ and PS_{120}-*b*-$P4VP_{20}$ are represented as PS_{24}-*b*-$P4VP_4$, PS_{36}-*b*-$P4VP_6$ and PS_{12}-*b*-$P4VP_2$, respectively.

1.3 The solubility parameters and interaction parameters. The solubility parameters (δ) of solvents at 313 K and different pressure are obtained from MD simulation performed by Materials Studio 2017 (BIOVIA), and the detailed procedures for calculating the solubility parameter were described in our previous work.[4] Firstly, according to the densities and mole fractions (Table S2) of CO_2-expanded toluene at different pressures, we constructed a box with side length of 26 Å and 3D periodic boundary conditions using the amorphous cell module. The force field COMPASS II [6, 7] was adopted. After geometry and energy optimization, 1000 ps MD simulations were performed using the forcite module under NVT ensemble (time step =1 fs) with the Nosé thermostat [8] to control temperature at 313 K. The final 500 ps simulations were used to collect data for analysis, to obtain the cohesive energy density (CED) through the CED analysis in amorphous cell module, and further obtain the solubility

parameters from equation (S8). Following the same procedures, the solubility parameters of bead M4-1 and M4-2 were acquired. Then according to equations (S5 and S6), the a_{ij} and χ_{ij} parameters of the system could be calculated. The repulsion parameters (a_{ij}) between pairwise beads are tabulated in Table 1 (main text). The solubility parameters of solvent beads, the beads of 4 and PS-*b*-P4VP are listed in Table S2, and the Flory-Huggins parameters (χ_{ij}) between pairwise beads are tabulated in Table S3.

$$\delta = \sqrt{CED} \tag{S8}$$

2. Experimental information

2.1 Materials, preparation of copolymer and self-assembled fluorescent composites (SAFCs). Tetrahydrofuran (THF, Beijing Chemical Reagents Co.) was distilled from sodium benzophenone ketyl under argon immediately prior to use. 4-vinyl pyridine (4VP, Alfa Aesar, Bingjing, 96%) and styrene (St, Tianjin Chemical Reagent, 98%) were distilled under reduced pressure, respectively, and stored in a refrigerator. 2, 2-Azobis (isobutyronitrile) (AIBN, Shanghai Chemical Reagents Co., purity 98%) was recrystallized from ethanol twice. Benzyl dithiobenzoate (BDTB) was prepared using the methold described by Chiefari et al.[9] Compound 4 was synthesized in our lab as reported.[10] The preparation of copolymer PS_{245}-*b*-$P4VP_{40}$ ($M_{n,PS\text{-}b\text{-}P4VP}$ = 29 900 g mol^{-1} with dispersity index (DI) =1.4, $M_{n,\,P4VP}$ = 4300 g mol^{-1}), and the SAFCs in CO_2-expanded toluene were described in the reference.[11] The SAFCs (with molar ratio R_M of 4VP to 4 = 690) were obtained from the mixture of 4 (1.0×10^{-5} mol L^{-1}) and PS_{245}-*b*-$P4VP_{40}$ (5 mg mL^{-1}) in CO_2-expanded toluene at 313.15 K and varied pressure for 72 h. The CO_2-expanded liquids (CXLs) pressures were selected to range from 3.00 to 6.50 MPa, and the SAFCs samples free of CO_2 (0.10 MPa) were prepared for comparison. Toluene for FL measurements was spectrophotometric grade (≥ 99.9%) from Sigma-Aldrich and used as received. CO_2 (99.95%) was provided by Zhengzhou Shuangyang Gas Co. (Zhengzhou, China).

2.1.1 Preparation of P4VP. In a typical polymerization procedure, an appropriate amount of 4-vinylpyridine (4VP), BDTB, and AIBN (with a molar ratio of 4VP/ BDTB /AIBN = 50-100/1/0.3) and THF were added into a 25 mL dry glass tube, followed by three freeze–vacuum–thaw cycles. The tube was sealed under vacuum and then immersed into an oil bath at 353.2 K with magnetic stirring. After reaction for 55 min, the tube was cooled to room temperature immediately. The polymer was dissolved with

certain amount of THF, and then precipitated by dropping the solution into anhydrous methanol, followed by filtration to obtain the dithiobenzoate-terminated poly(4-vinylpyridine) [P4VP-SC(S)Ph3. Repeat the dissolving-precipitation procedure three times, afterwards the product was dried in a vacuum oven for 24 h to achieve the P4VP-SC(S)Ph (yield: 72.8%).

2.1.2 Preparation of copolymer PS-*b*-P4VP. The synthesis of PS-*b*-P4VP by reversible addition-fragmentation chain transfer (RAFT) polymerization is described as follows. A prescribed amount of AIBN, P4VP-SC(S)Ph (as chains transfer agent, CTA), styrene (St) and DMF were successively added into a dry glass tube with a magnetic bar. After three freeze-vacuum-thaw cycles, the tube was sealed under vacuum and then immersed in a oil bath at 353.2 K. After the prescribed time (300 min), the tube was rapidly cooled down to room temperature. The copolymer was dissolved with certain amount of DMF, and then the copolymer was precipitated by adding the polymer solution into a mixture of petroleum ether and absolute diethyl ether (1:1, volume ratio). After filtration, the precipitation was extracted with toluene and hydrochloric acidic water (pH = 2) successively for 24 h in the Soxhlet extractor to remove the unreacted homopolymers. After being dried in a vacuum oven for 24 h, the block copolymer PS-*b*-P4VP was obtained. (yield of PS-*b*-P4VP: 40.7%)

The detailed polymerization reaction conditions, the number-average molecular weight (M_n) and dispersity index (M_w/M_n) of P4VP and PS-*b*-P4VP determined by GPC, as well as the block length ratio of PS/P4VP by ^{1}H NMR are tabulated in Table S4.

2.2 General information. FT-IR spectra were recorded on a NEXUS-470 spectrometer (Nicolet, USA). ^{1}H NMR spectra were obtained from a DRX-400 NMR instrument with tetramethylsilane as an internal standard. The number-average molecular weight and dispersity index (DI) of P4VP and PS-*b*-P4VP were determined by gel permeation chromatography (GPC, Waters 515 HPLC pump, Waters 2414 Refractive index detector; Waters Corporation, Milford, MA, USA). The morphology of the SAFC was characterized by transmission electron microscopy (TEM), using a JEOL JEM-2100 microscope with an accelerating voltage of 200 kV (JEOL Ltd, Tokyo, Japan). TEM samples were prepared by dropping solutions on carbon-coated copper grids, absorbing the solvent on filter paper, and evaporating the solvent at room temperature. The grid samples were stained with iodine vapors for 6 h to selectively stain the P4VP block to

enhance the contrast. Fluorescence (FL) measurements were performed on a Hitachi FL-4500 spectrofluorometer. The FL spectra were recorded with an excitation wavelength of 390 nm.

3. Bonding length and angles of H-bonds, and the pressure-responsive number of copolymer chains in particular types of micelles

Table S5 lists the DFT-calculated distances and angles involved in the hydrogen bonding between 4 and P4VP chains of the SAFCs. And the structural data of SAFCs micelles from DPD simulations are tabulated in Table S6, including the number of copolymer chains in particular types of micelles and typical contour length of the worm-like micelles.

Supplementary Tables

Table S1. Number of DPD beads (N_{DPD}) in a PS-*b*-P4VP/4/CO_2-toluene chain.

Type of beads	C_n [a]	Number of repeat units	N_{DPD}
S	9.9	240 (360 or 120)	24 (36 or 12) [b]
4VP	9.9	40 (60 or 20)	4 (6 or 2) [b]
M4-1 (phenol unit)			2
M4-2 (benzene unit)			5
T (CO_2-toluene)			1

[a] Characteristic ratio (C_n) calculated from the synthia module in Materials Studio software.

[b] Calculated from Eqn. (S7).

Table S2. Solubility parameters for beads of PS-*b*-P4VP and 4, and solvent beads at 313.2 K and varied pressures.

Species	Molar fraction of CO_2 [a]	Density [a] (g/cm^3)	Solubility parameter [d] $10^3(J^{1/2}*m^{-3/2})$
S			18.57 [c]
4VP			20.91 [c]
M4-1		1.0710 [b]	24.62
M4-2		0.8730 [b]	19.78
T (0.1 MPa)	0	0.8612	18.04
T (1.5 MPa)	0.1614	0.8690	17.74
T (3.0 MPa)	0.3105	0.8873	17.56
T (4.0 MPa)	0.4086	0.8980	17.43
T (4.5 MPa)	0.4606	0.9031	17.20
T (5.0 MPa)	0.5116	0.9077	17.08
T (5.5 MPa)	0.5761	0.9114	16.76
T (6.0 MPa)	0.6418	0.9125	16.45
T (6.5 MPa)	0.7144	0.9117	15.88

[a] Calculated from the Peng-Robinson Equation of State by the Aspen Plus V8.6;

[b] Calculated using Advanced Chemistry Development (ACD/Labs) Software V11.02;

[c] from Reference [12] ;

[d] Calculated from MD simulation via Materials Studio 2017 in this work.

Table S3. Flory-Huggins parameters x_{ij} between pairwise beads in CO_2-expanded toluene at 313 K .

	M4-1	M4-2	4VP	S
M4-1	0			
M4-2	8.85	0		
4VP	5.20	0.48	0	
PS	13.83	0.55	2.07	0
T (0.1 MPa)	16.36	1.14	3.11	0.11
T (1.5 MPa)	17.89	1.57	3.80	0.26
T (3.0 MPa)	18.84	1.86	4.24	0.39
T (4.0 MPa)	19.54	2.09	4.58	0.49
T (4.5 MPa)	20.81	2.52	5.20	0.71
T (5.0 MPa)	21.49	2.76	5.54	0.84
T (5.5 MPa)	23.35	3.45	6.51	1.24
T (6.0 MPa)	25.23	4.19	7.52	1.70
T (6.5 MPa)	28.86	5.75	9.56	2.73

Table S4. Polymerization of P4VP and PS-*b*-P4VP at 353.2 K and different reaction conditions

Sample	Time (min)	Initiator	4VP/BDTB/ AIBN (molar ratio)	M_n [a]	M_w/ M_n [a]	PS/P4VP [b]	Yield (%)
P4VP	55	AIBN	50-100: 1: 0.3	4300	1.36		72.8
PS-*b*-P4VP	300	AIBN		29900	1.39	6:1	40.7

[a] The number-average molecular weight (M_n) and dispersity index (M_w/ M_n) of P4VP and P4VP-*b*-PS were determined by GPC in DMF at 313.2 K, flow rate 1ml/min.

[b] Block length ratio of PS/P4VP based on ^{1}H NMR analysis, $CDCl_3$ as solvent and tetramethylsilane (TMS) as internal standard.

Table S5. The optimized structural data involved in hydrogen bonding for the SAFCs.

Structure	Bond lengths (Å)	Bond angles (°)	Bond lengths (Å) [Ref]
H-bonds (a)	1.744	175.5	1.78-1.79 [13]
H-bonds (b)	1.763	175.0	

Table S6. The structural information of SAFCs micelles or aggregates from DPD simulations [a]

Pressure (MPa)	Morphology	Number of copolymer chains [b]	Length of rods [c] (Å)
0.1	aggregated clusters	11 (0.02)	
3.0	spheres	28 (0.42)	
4.0	spheres	44 (0.83)	
	worm-like micelles	53 (0.85)	115.0 (0.6)
4.8	spheres	62 (1.25)	
	worm-like micelles	141 (2.51)	201.0 (3.2)
5.5 - 6.5	vesicle-like micelles	323 (5.09)	

[a] The SAFCs of copolymer blend PS-*b*-P4VP/4 in CO_2-expanded toluene (313.2 K/varied pressure), copolymer blends PS_{240}-*b*-$P4VP_{40}$ with dispersity index = 1.4 (mass fraction of 75% PS_{240}-*b*-$P4VP_{40}$ +10% PS_{360}-*b*-$P4VP_{60}$ +15% PS_{120}-*b*-$P4VP_{20}$).

[b] The average number of copolymer chains per unit micelle, data given in parentheses refer to the corresponding standard deviation (SD) data.

[c] Data given in parentheses refer to the corresponding SD data.

Supplementary Figures

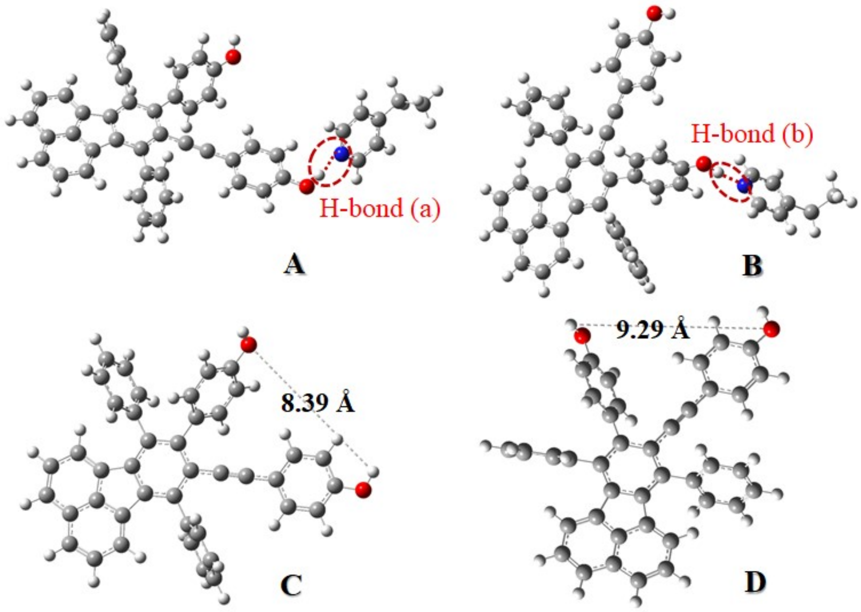

Figure S1. Optimized structures for the potential hydrogen bonds. Atom O in red, N in blue, C in gray and H in white. (A) H-bond (a), the hydrogen bond between ethynylphenolic OH group of 4 and nitrogen atom of 4VP; (B) H-bond (b) between phenolic OH of 4 and nitrogen atom of 4VP; (C) and (D) illustrate the impossible intra-molecular hydrogen bonds in molecule 4.

References

1. P. J. Hoogerbrugge and J. M. V. A. Koelman, *Europhys. Lett.*, 1992, **19**, 155-160.
2. J. M. V. A. Koelman and P. J. Hoogerbrugg, *Europhys. Lett.*, 1993, **21**, 363-368.
3. R. D. Groot and P. B. Warren, *J. Chem. Phys.*, 1997, **107**, 4423-4435.
4. J. Wei, Y. Li, L. Su, C. Feng and H. Li, *Comput. & Appl. Chem.*, 2020, **37**, 23-29.
5. M. Liao, H. Liu, H. Guo and J. Zhou, *Langmuir*, 2017, **33**, 7575-7582.
6. H. Sun, P. Ren and J. R. Fried, *Comput. Theor. Polym. Sci.*, 1998, **8**, 229-246.
7. A. Maitia and S. McGrother, *J. Chem. Phys.*, 2004, **120**, 1594-1601.
8. S. Nose, *J. Chem. Phys.*, 1984, **81**, 511-519.
9. J. Chiefari, Y. K. Chong, F. Ercole, J. Krstina, J. Jeffery, T. P. T. Le, R. T. A. Mayadunne, G. F. Meijs, C. L. Moad, G. Moad, E. Rizzardo and S. H. Thang, *Macromolecules*, 1998, **31**, 5559-5562.
10. Y. Guo, X. Yu, W. Xue, S. Huang, J. Dong, L. Wei, M. Maroncelli and H. Li, *Chem. Eng. J.*, 2014, **240**, 319-330.
11. X. Cheng, S. Huang, H. Li, N. An, Q. Wang and Y. Li, *RSC Adv.*, 2016, **6**, 4545-4551.
12. C. Zhang, J. Hu, D. Chen, Y. Zhu, Y. Fan and Y. Liu, *Polymer*, 2012, **53**, 4718-4726.
13. P. K. Biswas, A. Goswami, S. Saha and M. Schmittel, *Chem. Eur. J.*, 2020, **26**, 14095-14099.
